# Case Report: Bilateral pes planovalgus: an uncommon pediatric orthopedic presentation of Charcot–Marie–Tooth disease type 4B1

**DOI:** 10.3389/fped.2026.1810832

**Published:** 2026-09-30

**Authors:** Hamad Ali Alkhunayfir, Rahaf Hamad Alrasheed, Abdelhamid Elshahid Hassan, Bader Faiz Alsubaie

**Affiliations:** 1 Orthopedic Department, King Fahad Medical City, Riyadh, Saudi Arabia; 2 College of Medicine, King Saud University, Riyadh, Saudi Arabia

**Keywords:** Charcot–Marie–Tooth disease type 4B1, flatfoot, MTMR2 gene mutation, pediatric neuropathy, pes planovalgus deformity

## Abstract

Charcot–Marie–Tooth (CMT) disease is an inherited motor and sensory neuropathy that typically presents with cavovarus foot deformity. Pes planovalgus is uncommon in CMT and, while noted incidentally in multicenter cohort studies of CMT4B subtypes, has not been described as the predominant orthopedic manifestation in a published case report of genetically confirmed CMT4B1. We report the case of a 7-year-old girl who presented with progressive painful bilateral pes planovalgus, hallux valgus, frequent falls, and gait imbalance, despite otherwise normal developmental milestones. Neurological examination revealed globally reduced deep tendon reflexes, absent ankle jerks bilaterally, and distal sensory impairment in a length-dependent distribution. Electrodiagnostic studies demonstrated a severe mixed demyelinating and axonal polyneuropathy with completely absent distal motor and sensory responses in the lower limbs. Genetic analysis identified a pathogenic MTMR2 variant, confirming the diagnosis of autosomal recessive CMT type 4B1. Weight-bearing radiographic evaluation demonstrated bilateral collapse of the medial longitudinal arch, forefoot abduction, and hallux valgus deformity. The patient was managed in a multidisciplinary setting with physiotherapy, occupational therapy, and orthotic support. Conservative management resulted in satisfactory clinical improvement on the left side; however, the right foot demonstrated persistent pain and functional limitation despite adequate conservative treatment. Surgical correction of the right foot has been planned, including Evans lateral column lengthening osteotomy, medial talonavicular capsule plication, and Tendo-Achilles lengthening, with possible medial midfoot closed wedge osteotomy and flexor tendon release. This case illustrates phenotypic variability within CMT4B1. In contrast to her older brother, who carries the same MTMR2 variant and developed the classic cavovarus deformity, our patient presented with early progressive pes planovalgus, broadening the recognized phenotypic spectrum of this rare subtype. Recognizing this variant presentation is essential to avoid misdiagnosis and to prompt timely neurological, genetic, and orthopedic evaluation in children presenting with painful flatfoot and signs of peripheral neuropathy.

## Introduction

Charcot–Marie–Tooth (CMT) disease is an inherited neuromuscular disorder and the most common hereditary cause of cavus foot deformity (1). It is classically characterized by progressive distal extremity weakness, predominantly affecting the anterior and lateral compartment muscles of the leg and the intrinsic muscles of the foot, accompanied by sensory loss. This muscle imbalance typically results in a cavoadductovarus deformity with digital contractures, presenting clinically as pes cavus and claw toes (2). Gait disturbance is among the most functionally significant consequences of this progressive neuropathy, often leading to reduced mobility, frequent falls, and decreased quality of life (3). Diagnosis is established through neurophysiological studies and genetic testing, with the most common form involving PMP22 gene duplication on chromosome 17 in an autosomal dominant inheritance pattern (4, 5).

However, not all CMT cases follow this classic pattern. CMT type 4B1 (CMT4B1) is a rare autosomal recessive demyelinating form caused by pathogenic mutations in the MTMR2 gene. It typically presents in early childhood with a severe and rapidly progressive neuropathy, often with a more aggressive disease trajectory compared with the dominant CMT1 subtypes (5, 6). Reaching an accurate genetic diagnosis in such cases is essential, as emerging treatments and clinical trials increasingly target specific CMT subtypes and rely on precise molecular characterization (7). While pes planus has been noted among patients in multicenter CMT4B studies (6), only a few individual cases have been published, and the phenotypic spectrum of CMT4B1 remains incompletely characterized. Recent studies have further highlighted the variability of foot deformity morphology across CMT subtypes (8), underscoring the importance of reporting atypical presentations. In this case report, we present a 7-year-old girl with genetically confirmed CMT4B1 who developed severe bilateral pes planovalgus, supported by radiographic and electrophysiological findings, to further characterize the phenotypic variability of this rare CMT subtype.

## Case presentation

A 7-year-old Saudi girl was referred from another hospital to our multidisciplinary pediatric clinic for evaluation because of progressive bilateral pes planovalgus, hallux valgus, frequent falls, and gait imbalance. She was medically free and had reached all developmental milestones at the appropriate age. Her mother first noticed right-sided foot deformity at the age of six, which gradually progressed to involve both feet. Over time, she developed tiptoe walking, frequent falls, and difficulty running and climbing stairs without support. She also had difficulties with fine motor skills, particularly with pencil grip and handwriting. Given these motor challenges, her cognitive and academic performances were age-appropriate.

She was born full-term by cesarean section due to breech presentation, with no perinatal complications. Her parents are first cousins, and her 16-year-old brother has genetically confirmed CMT4B1 caused by the same familial MTMR2 variant; in contrast to our patient, he developed a cavovarus foot deformity that required corrective surgery. Her 12-year-old sister is healthy.

On examination, the patient was alert, interactive, and non-dysmorphic with no dysmorphic facial features. Cranial nerve examination was unremarkable. Muscle tone was normal in both upper and lower limbs. There was mild asymmetry in lower limb muscle bulk, with the right calf visibly thinner than the left, suggesting early distal muscle atrophy. Manual muscle testing revealed mild weakness of ankle dorsiflexion and plantarflexion bilaterally, more pronounced on the right, with relatively preserved proximal lower limb strength. Upper limb strength was grossly intact, though mild fine motor difficulty was noted clinically. Deep tendon reflexes were globally reduced, graded +2 in the upper limbs and +1 in the lower limbs, with absent ankle jerks bilaterally. Sensory examination revealed reduced light touch and pinprick sensation in a length-dependent stocking distribution in the lower limbs. Gait assessment revealed bilateral flexible pes planovalgus deformity, more severe on the right side, associated with forefoot abduction and gastrocnemius tightness. Tiptoe walking was observed, and the patient was unable to perform heel walking bilaterally. There was no scoliosis, cerebellar dysfunction, tremor, or upper motor neuron signs.

She was evaluated by the neurology team. Nerve conduction studies revealed absent responses in the bilateral peroneal (extensor digitorum brevis) and tibial (abductor hallucis brevis) motor nerves, prolonged distal latencies in the median nerves, and markedly reduced conduction velocities across both upper and lower limbs. Sensory studies showed absent median and ulnar responses and reduced sural amplitudes. Needle electromyography demonstrated increased motor unit amplitude and duration with evidence of denervation in the distal lower limb muscles (Table 1). These findings indicated a mixed demyelinating and axonal polyneuropathy.

**Table 1:** Nerve conduction study results.

Nerve	Site	Latency (ms)	Normal (ms)	Amplitude	Normal	CV (m/s)	Normal (m/s)	Interpretation
Motor studies (amplitude in mV)								
Left fibular (EDB)	Ankle	NR	<6.1	NR	>2.0	NR	>44	Absent—severely abnormal
Right fibular (EDB)	Ankle	NR	<6.1	NR	>2.0	NR	>44	Absent—severely abnormal
Tibial (AHB)	Ankle	NR	<5.8	NR	>2.0	NR	>40	Absent—severely abnormal
Median (APB)	Wrist/elbow	6.1	<4.0	2.5	>4.0	14	>48	Prolonged latency, severely reduced CV
Ulnar (ADM)	Wrist/elbow	4.4	<3.6	3.0	>4.0	16	>48	Reduced CV—severely abnormal
Sensory studies (amplitude in µV)								
Left median	Wrist–digit	NR	<3.5	NR	>10	NR	>48	Absent—severely abnormal
Right median	Wrist–digit	NR	<3.5	NR	>10	NR	>48	Absent—severely abnormal
Left ulnar	Wrist–digit	NR	<3.2	NR	>10	NR	>48	Absent—severely abnormal
Right ulnar	Wrist–digit	NR	<3.2	NR	>10	NR	>48	Absent—severely abnormal
Left sural	Calf–lat. malleolus	3.6	<4.0	6.0	>6	35	>40	Reduced amplitude and CV—abnormal
Right sural	Calf–lat. malleolus	3.7	<4.0	6.1	>6	35	>40	Reduced amplitude and CV—abnormal

NR, no response; CV, conduction velocity; EDB, extensor digitorum brevis; AHB, abductor hallucis brevis; APB, abductor pollicis brevis; ADM, abductor digiti minimi. Motor amplitudes are reported in mV and sensory amplitudes in µV.

Age-adjusted normal values for a 7-year-old child: peroneal motor conduction velocity >44 m/s; median motor conduction velocity >48 m/s; median motor distal latency <4.0 ms; sural sensory amplitude >6 µV; median sensory amplitude >10 µV; sural conduction velocity >40 m/s. All values were significantly outside normal limits, consistent with a severe demyelinating neuropathy with prominent sensory involvement.

Following these results, the patient was referred to the genetics team. Targeted Sanger sequencing confirmed a homozygous pathogenic frameshift variant in MTMR2 [NM_016156.5:c.1736_1745delinsCC, p.(Tyr579Serfs*22)], classified as pathogenic according to American College of Medical Genetics and Genomics (ACMG) criteria, thereby establishing the diagnosis of autosomal recessive Charcot–Marie–Tooth disease type 4B1 (CMT4B1). The same variant was subsequently identified in her affected older brother, consistent with segregation within this consanguineous family.

Radiographic evaluation demonstrated bilateral pes planovalgus deformity. The lateral view showed collapse of the medial arch (Figure 1A), while the anteroposterior view showed forefoot abduction and hallux valgus deformity (Figure 1B). Weight-bearing lateral radiographs further confirmed the deformity under load (Figure 1C).

**Figure 1:**
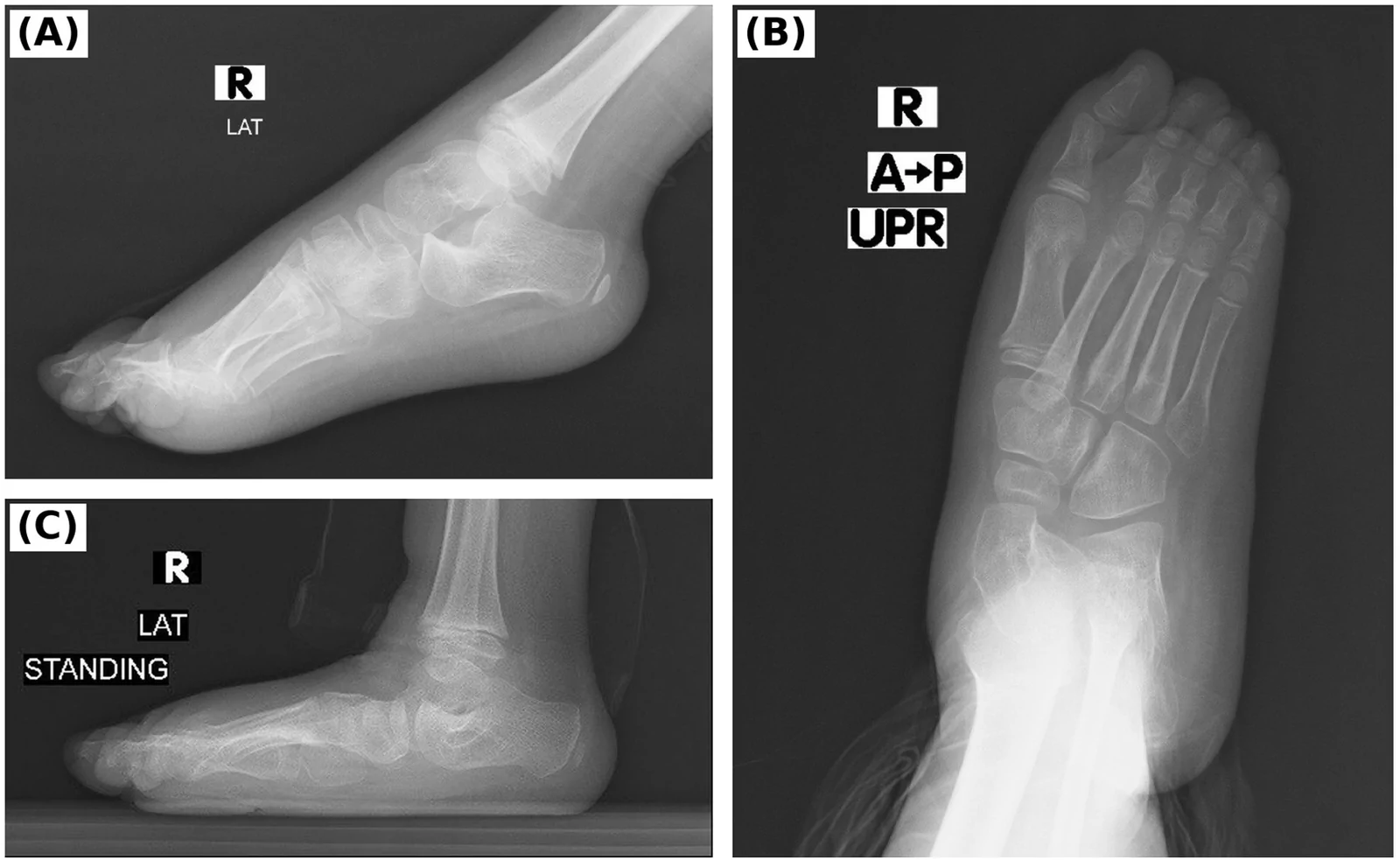
Weight-bearing radiographs of the right foot. **(A)** Lateral radiograph showing collapse of the medial longitudinal arch; **(B)** anteroposterior radiograph demonstrating forefoot abduction and hallux valgus; and **(C)** weight-bearing lateral radiograph confirming pes planovalgus under load.

After diagnosis, the patient was referred to physiotherapy, occupational therapy, and orthotics. Physiotherapy documented weakness in the calves and hamstrings, tiptoe gait, and reduced endurance, while occupational therapy identified difficulties with handwriting, dressing, and stair climbing and recommended school accommodations. Orthotic devices were prescribed and adjusted accordingly; orthotic intervention has been reported to play an important role in managing deformity and improving function in CMT-related foot conditions (9).

Orthopedic evaluation was performed for surgical planning. Given the severity of the right foot deformity, the surgical team recommended Evans lateral column lengthening osteotomy, medial talonavicular capsule plication, and Tendo-Achilles lengthening, with possible medial midfoot closed-wedge osteotomy and flexor tendon release, depending on intraoperative assessment findings. The left foot continues to be managed conservatively with orthotic support and physiotherapy. Surgery is planned for later this year, at which point the patient will be approximately 9 years of age.

At last follow-up, the patient remained ambulatory with minimal assistance and continued to attend school. She is currently awaiting right foot corrective surgery. The overall episode of care is summarized in Figure 2.

**Figure 2:**
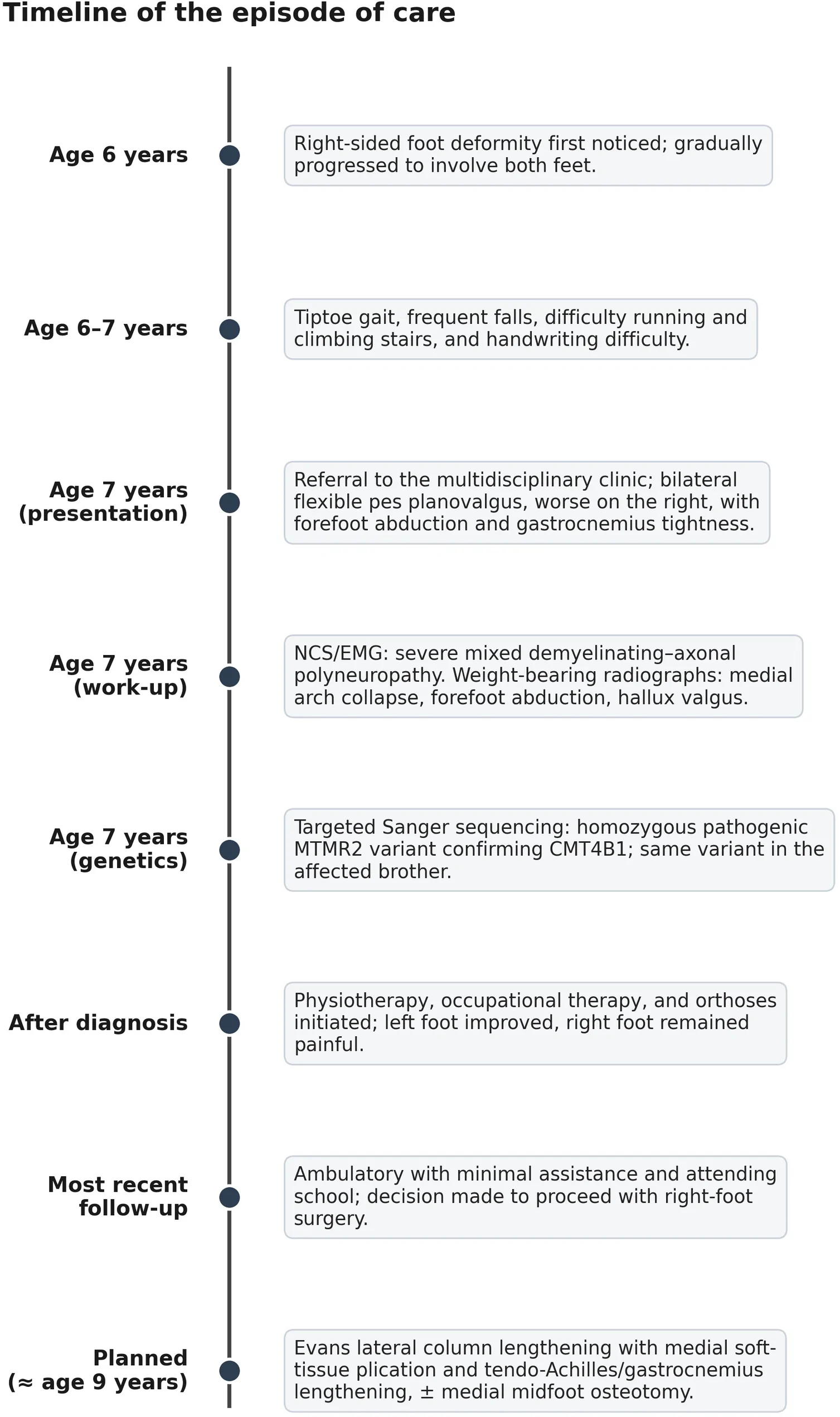
Timeline of the episode of care, from initial presentation at age 6 through diagnostic work-up, genetic confirmation of CMT4B1, conservative management, and planned right-foot reconstructive surgery.

## Discussion

CMT disease is considered the most common inherited motor and sensory neuropathy (10), with a prevalence of around 1 in 2,500 in some populations (4). It is classified into several subtypes based on genetic mutations and clinical features; these include the demyelinating forms (CMT1), axonal forms (CMT2), X-linked types (CMTX), and the rarer autosomal recessive demyelinating form (CMT4), which often presents earlier and tends to follow a more severe, rapidly progressive course than the dominant CMT1 types (5). This aligns with our case, which involves CMT4 caused by MTMR2 mutations and represents a foot phenotype not previously described in a published case report of genetically confirmed CMT4B1.

Harding and Thomas described how attempts to classify these neuropathies were complicated by the wide variability in clinical features, historical confusion, and difficulty distinguishing phenotypes (11). As understanding of CMT has expanded over the past several decades, classification systems have been reshaped from the disease’s original introduction in the 1970s. Rebelo et al. described two patients as cases of CMT4 (12). Reaching an accurate genetic diagnosis is essential since treatments and trials focus on specific CMT types and rely on knowing the exact mutation (7).

A multicenter study by Pareyson et al. found that patients with CMT4 had very slow nerve conduction and clear signs of demyelination, underscoring the importance of nerve studies in diagnosis (6). Consistent with these findings, our patient's electrophysiological studies showed a severe demyelinating pattern (Table 1): Motor nerve conduction responses in the distal lower limbs were absent, while median and ulnar motor studies showed reduced conduction velocities (14–16 m/s). Sensory studies (Table 1) revealed absent median and ulnar sensory potentials, with only the sural nerves showing recordable responses at significantly reduced amplitudes, characteristic of the slowing and sensory involvement typically seen in CMT4 subtypes.

Children presenting with bilateral cavovarus feet have a high likelihood of underlying CMT, with 78% receiving a confirmed diagnosis and the probability increasing to 91% in those with a positive family history (13). The classic deformity arises from weakness of the tibialis anterior, peroneus brevis, and intrinsic foot muscles, combined with relatively preserved strength of their antagonists, the peroneus longus and tibialis posterior, leading to progressive cavus, forefoot equinus, and calcaneal varus, as described by Mann and Missirian in their classic study on the pathophysiology of CMT (2). Recent studies using advanced imaging have further characterized the structural differences in foot morphology between demyelinating and axonal CMT subtypes, highlighting the variability of deformity patterns across the disease spectrum (8). CMT is the most common diagnosis associated with cavus foot (1). However, our patient presented with bilateral pes planovalgus (Figure 3), similar to Miller's study of a CMT1A patient who developed progressive pes planovalgus rather than the typical cavovarus morphology (14). Unlike that CMT1A case, our patient was diagnosed with autosomal recessive CMT4B1 caused by an MTMR2 mutation. While pes planus has been noted among patients in multicenter CMT4B studies (6), to our knowledge no prior case report has described bilateral progressive pes planovalgus as the predominant orthopedic manifestation in a genetically confirmed CMT4B1 patient. This conclusion is based on a literature search of PubMed, Embase, and Google Scholar (from inception to June 2026) using the terms “CMT4B1,” “MTMR2,” “pes planovalgus,” “pes planus,” and “flatfoot,” alone and in combination, which identified no previously published case report describing bilateral pes planovalgus as the predominant orthopedic manifestation of genetically confirmed CMT4B1.

**Figure 3:**
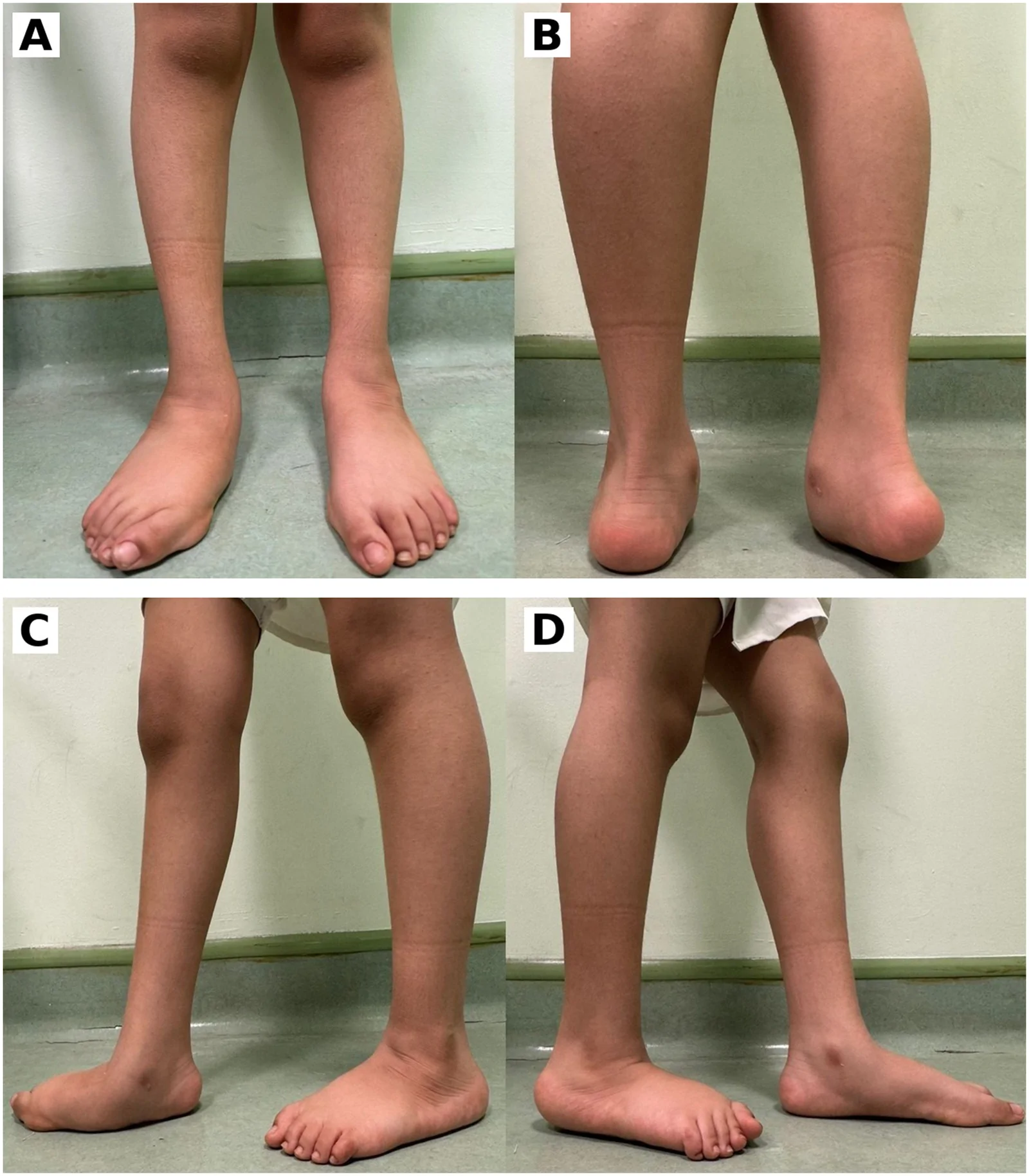
Clinical photographs of the lower limbs. **(A)** Anterior standing view showing bilateral pes planovalgus with medial arch collapse and forefoot abduction; **(B)** posterior standing view demonstrating bilateral hindfoot valgus; and **(C,D)** lateral standing views showing loss of the medial longitudinal arch.

The development of pes planovalgus rather than the expected cavovarus deformity in this patient warrants detailed biomechanical consideration. The classical cavovarus deformity in CMT results from a predictable pattern of muscle imbalance: weakness of the tibialis anterior, peroneus brevis, and intrinsic foot muscles, combined with relatively preserved strength of the peroneus longus and tibialis posterior, produces progressive forefoot equinus, first-ray plantarflexion, and calcaneal varus (2). However, when this pattern of selective muscle denervation is altered—particularly when the tibialis posterior itself is significantly weakened—the biomechanical forces that normally drive cavus formation are disrupted, and a planovalgus deformity may develop instead.

In our patient, the electrophysiological findings demonstrated a severe mixed demyelinating and axonal polyneuropathy with completely absent distal motor and sensory responses in the lower limbs, indicating profound and diffuse distal denervation rather than the selective muscle weakness pattern typically seen in CMT1A. This severity and non-selective pattern of denervation may have resulted in global weakness of all foot and ankle stabilizing muscles, including the tibialis posterior, removing the primary dynamic stabilizer of the medial longitudinal arch and allowing progressive planovalgus deformity to develop. In this context, the gastrocnemius tightness observed clinically may have further contributed to the deformity by generating a deforming force that, in the absence of adequate dynamic stabilization, promoted forefoot abduction and hindfoot valgus rather than the equinus and varus seen in classic CMT.

This proposed mechanism differs from the explanations offered in Miller's CMT1A case (14), where tibialis posterior weakness, early disease stage before cavus progression, and possible ligamentous laxity were suggested as contributing factors. In our patient, there was no clinical or radiographic evidence of ligamentous laxity, no signs of early cavus formation, and the electrophysiological severity far exceeded that reported in CMT1A, with completely absent rather than merely slowed nerve conduction responses. This raises the possibility that the planovalgus phenotype in CMT4B1 is driven, at least in part, by profound and non-selective distal denervation rather than the selective muscle imbalance that underlies the classic cavovarus deformity. An important consideration in this family is that the patient's older brother carries the same MTMR2 variant yet developed a cavovarus deformity requiring surgical correction. This intrafamilial discordance indicates that the CMT4B1 genotype alone does not determine foot morphology, and that additional factors—including the patient's young age, the current flexibility of the deformity, and the stage of disease at the time of assessment—are likely to contribute. It also raises the possibility that the present planovalgus represents an early, age-dependent phase that could evolve toward a cavovarus deformity with skeletal maturation, as has been described in pediatric CMT (15). Longitudinal follow-up will therefore be essential to determine whether the planovalgus persists or converts over time. Accordingly, the present report is best interpreted as expanding the recognized phenotypic spectrum of CMT4B1, rather than defining a fixed, subtype-specific deformity.

Furthermore, the autosomal recessive CMT4 subtypes, including CMT4B1, are known to present earlier and follow a more rapidly progressive disease course compared to the dominant CMT1 subtypes (5). This accelerated progression may result in simultaneous denervation of multiple muscle groups before any selective imbalance can establish itself, potentially explaining why the foot deformity in CMT4B1 does not follow the stepwise progression toward cavus seen in milder subtypes. The combination of early onset, rapid progression, severe mixed demyelinating and axonal neuropathy, and profound distal denervation likely contributes to the development of a planovalgus rather than cavovarus deformity in this rare CMT subtype.

Treatment options in CMT disease depend on factors such as the patient's age, the flexibility of the hindfoot, and how quickly the deformity is progressing, as the goal is to balance risks, benefits, and expected functional outcomes according to individual needs (16). Surgical management of CMT-related foot deformities usually involves a combination of soft-tissue balancing, tendon transfers, and osteotomies, with arthrodesis reserved for rigid or severe deformities (17). Reconstructive techniques such as calcaneal osteotomy, first-ray dorsiflexion osteotomy, and tendon transfers are considered the mainstay surgical options for flexible deformities in CMT (1). However, our patient presented with a progressive pes planovalgus deformity, which requires a different treatment approach. For flexible planovalgus deformity, standard pediatric reconstruction techniques—such as lateral column lengthening (Evans osteotomy), Cotton osteotomy, gastrocnemius recession, and medial soft tissue plication—are well established and widely used in neuromuscular flatfoot correction (18). The left side was managed conservatively with medial arch support and physiotherapy aimed at strengthening the tibialis posterior muscle and stretching the gastro–soleus complex to reduce dynamic collapse and improve gait stability, consistent with current evidence supporting tailored physiotherapy programs as essential for managing gait disturbances in CMT (10). Given the more severe deformity on the right side, surgical correction is planned using standard surgical techniques for flexible pes planovalgus. As surgery is planned but not yet performed, the intended goals of the surgical intervention are to relieve pain, achieve a plantigrade foot, restore normal foot biomechanics, and preserve hindfoot and midfoot motion as much as possible to maintain long-term function. Miller's case involved a child with CMT1A and a rigid pes planovalgus deformity, who ultimately required triple arthrodesis with gastrocnemius recession due to pain, midfoot collapse, and significant functional limitations associated with the stiffness of the deformity. In contrast, our patient presents with a flexible deformity at a younger age, and the planned surgical approach aims to correct the deformity while avoiding early fusion, in order to preserve hindfoot and midfoot motion and maintain long-term functional capacity. The surgical plan has been designed based on the current deformity characteristics and the established principles of flexible planovalgus correction in pediatric neuromuscular conditions (18). This contrast highlights the importance of tailoring surgical strategies to deformity type, severity, and flexibility, particularly in growing children with progressive inherited neuropathies.

In conclusion, this case expands the recognized phenotypic spectrum of CMT4B1 by documenting bilateral progressive pes planovalgus as the predominant orthopedic manifestation in a patient with genetically confirmed autosomal recessive CMT4B1 caused by an MTMR2 mutation. This finding is significant because it challenges the conventional understanding that pes cavovarus is the universal hallmark deformity of CMT disease. This differentiation has important implications for both diagnosis and management. Early recognition of this presentation can prevent misdiagnosis. Reporting such atypical manifestations expands the clinical understanding of CMT4 subtypes and underscores the importance of integrating genetic, electrophysiologic, and orthopedic assessments when managing complex pediatric neuropathies.

This report has several limitations. As a single case, the findings cannot be generalized to all patients with CMT4B1. The planned surgery has not yet been performed, so intraoperative findings and postoperative outcomes are not available, and follow-up to date is limited. Longer-term follow-up will be needed to determine whether the planovalgus deformity persists or evolves over time. In addition, formal weight-bearing radiographic angles were not available, and the radiographic diagnosis was based on qualitative assessment of arch collapse and forefoot abduction.

## Conclusions

This case expands the recognized phenotypic spectrum of CMT4B1 by demonstrating that bilateral progressive pes planovalgus, rather than the classical cavovarus deformity, can be the predominant orthopedic manifestation in a child with genetically confirmed autosomal recessive MTMR2 mutation. The severe mixed demyelinating and axonal neuropathy observed in this patient, with completely absent distal motor and sensory responses, likely underlies a distinct biomechanical pathway involving global distal denervation and tibialis posterior weakness, driving planovalgus rather than cavus deformity formation.

This atypical presentation carries important diagnostic and clinical implications. Clinicians evaluating children with painful progressive flatfoot should consider CMT and related inherited neuropathies in the differential diagnosis, particularly when accompanied by signs of peripheral neuropathy, a positive family history, or consanguinity. Early neurological and genetic evaluation is essential to establish an accurate diagnosis, guide appropriate management, and enable timely genetic counseling for affected families.

From an orthopedic perspective, treatment must be individualized based on deformity type, severity, and flexibility rather than following protocols designed exclusively for cavovarus correction. In flexible planovalgus deformity, joint-preserving reconstructive techniques should be prioritized, particularly in young growing children, to maintain long-term mobility and function. Multidisciplinary care integrating neurology, genetics, orthopedics, physiotherapy, and occupational therapy remains the cornerstone of management for this complex pediatric condition.

Reporting such atypical manifestations is essential to refine the clinical understanding of CMT4 subtypes, challenge assumptions about universal deformity patterns in inherited neuropathies, and ultimately improve diagnostic accuracy and patient outcomes in this challenging population.

## Data Availability

The raw data supporting the conclusions of this article will be made available by the authors, without undue reservation.
